# Self-medication practices in Ethiopia: An umbrella review protocol

**DOI:** 10.1371/journal.pone.0300131

**Published:** 2025-02-27

**Authors:** Getahun Fetensa, Eshetu Ejeta, Tariku Tesfaye Bekuma, Feyiso Bati, Edosa Amante, Jaleta Bulti, Saro Abdella, Yosef Gebreyohannes, Sabit Ababor, Abera Botore, Dabesa Gobena, Mirkuzie Woldie, Minyahil Tadesse Boltena, Tadesse Tolossa, Tafese Dejene, Zewdie Birhanu, Moranker Sudakar

**Affiliations:** 1 Department of Health Behavior and Society, Faculty of Public Health, Institute of Health, Jimma Medical Center, Jimma University, Jimma, Ethiopia; 2 Ethiopian Public Health Institute Addis Ababa, Addis Ababa, Ethiopia; 3 Center for Evidence-synthesis, Support, and Development in Africa (CESDA), PLC, Addis Ababa, Ethiopia; 4 Department of Public Health, College of Medical and Health Sciences, Ambo University, Ambo, Ethiopia; 5 Department of Public Health, Faculty of Public Health, Institute of Health, Department of Population and Family Health, Jimma University, Jimma, Ethiopia; 6 Department of Public Health, Institutes of Health Sciences, Wollega University, Nekemte, Ethiopia; 7 Knowledge Translation Directorate, Ethiopian Public Health Institute, Addis Ababa, Ethiopia; 8 Public Health Emergency Management and Health Research Directorate, Oromia Health Bureau, Addis Ababa, Ethiopia; 9 Fenot Project, School of Population and Public Health, University of British Columbia, Addis Ababa, Ethiopia; 10 Ethiopian Evidence Based Health Care Centre: A Joanna Briggs Institute Center of Excellence, Faculty of Public Health, Institute of Health, Jimma University, Jimma, Ethiopia; 11 Knowledge Translation Division, Knowledge Management Directorate, Armauer Hansen Research Institute, Ministry of Health, Addis Ababa, Ethiopia; 12 Deakin University, Deakin Health Economics, School of Health and Social Development, Institute for Health Transformation, Geelong, Australia; 13 Department of Obstetrics and Gynecology, Dire Dawa University, Dire Dawa, Ethiopia; Haramaya University, ETHIOPIA

## Abstract

**Background:**

Self-medication is the practice of obtaining and using drugs without proper guidance or supervision. It can involve various behaviours that may harm individuals and society. Self-medication can cause serious health and economic problems for countries and healthcare systems. Therefore, current review aimed at examining self-medication practices in Ethiopia.

**Methods:**

This umbrella review will consider all systematic reviews that include self-medication practices of adult age greater than 18 years in Ethiopia. Study protocols, papers other than systematic reviews, papers not reporting on self-medication practices, and papers published in languages other than English will be excluded from the review. MEDLINE, Embase, and the Cochrane Database of Systematic Reviews will be searched. Two reviewers will screen the titles and abstracts against the eligibility criteria. Data extraction will be performed by 2 independent reviewers on the reviews selected for inclusion. The characteristics of studies like author name, year published, databases searched, number of studies/patients included, and self-medication practices will be extracted. The quality of included studies will be reported using the JBI critical appraisal checklist for systematic reviews and research syntheses. A summary of the extracted data will be presented in tabular format and a narrative synthesis will be performed on the collected systematic reviews that meet the inclusion criteria.

**Discussion:**

This protocol is expected to bring pooled evidence on self-medication practice among different population groups like pregnant mothers, adult population, students, and general population. Evidence from this review will be used to tackle current global problem related with anti-microbial resistance. Therefore, our review will call for government and non-government interventions in reducing current challenge of global issue related with anti-microbial resistance in resource limited country like Ethiopia.

**Systematic review registration number:**

PROSPERO CRD42023182552

## Introduction

Self-medication refers to the method of acquiring the drug in the absence of a particular healthcare provider and the source of the medication [1]. Antibiotic resistance may develop and spread faster due to this improper use of drugs [2]. It can be extended to the act of sharing and lending medications as types of self-medication, along with other intriguing ideas like self-care, disregarding a prescription, reusing stored medications, and not taking them as directed by health professionals [1]. People rely on their own experience and information from trustworthy sources such as chemists, pharmacists, and other health professsionals are not considered [3].

Self-medication can have detrimental effects on society and healthcare systems in the short- and long-term, costing nations a tremendous amount of money [4]. It is a source for a worldwide expanding phenomenon that poses a threat to public health due to currently existing problems like antibiotic resistance, the potential for harmful side effects, drug interactions, and disease masking [1]. Furthermore, it has a cumbersome contribution to increasing the up growth and expansion of antibiotic resistance [2]. Nearly half of the Ethiopian university students practiced self-medication, which is very high given the current global health problem of anti-microbial resistance increment [5–7]. Self-medication was also reported to be commonly practiced among the adult population [8] and pregnant women [9,10].

Self-medication practice varies widely because of different factors such as where people live, their cultural background, how easy it is to get health care, and accessibility of the pharmacy [11]. Self-medication is mostly used for affordability, speed of relief, and reduced time commitment [3]. In addition, factors, like its affordability [11], a previous history and the minor nature of the disease were significantly associated for self-medication practice [4]. Furthermore, it is highly related to self-care. Self-care is the ability of individuals, families, and communities to promote health, prevent disease, maintain health, and cope with illness and disability with or without the support of a health worker [12]. Although self-medication can be considered a kind of self-care, there are some situations where this definition cannot be limited to the realm of health [1]. Currently, 3.6 billion people lack access to essential health services [13]. WHO recommends self-care interventions for every country and economic setting, as a critical path to reaching universal health coverage, promoting health, keeping the world safe, and serving the vulnerable [14].

Self-medication has piqued the curiosity of public health experts worldwide. Most of the drugs commonly used for self-medication in Africa are anti-malaria, antibacterial, and analgesics. Improper drug usage could contribute to the rise in drug resistance (antibacterial& antimalarial) [15]. People do not trust commercial sources such as health magazines and advertisements for self-medication. Self-medication is also not influenced by the patients’ education level. The main reasons why people practice self-medication are affordability, quick relief and saving time [3].

It is very crucial to control the supply of antibiotics by enforcing the current regulations strictly and/or adopting new ones, and by training and educating pharmacy staff (such as pharmacists and pharmacy assistants) on how to use antibiotics responsibly and the significance of antimicrobial stewardship [16].

Even though the positive aspects of self-medication related to primary healthcare should be recognized in low-income countries, the risk may outweigh the benefit if the current trend continues [17]. People in developing countries like Ethiopia not only use drugs they aren’t prescribed but also prescribed drugs without being supervised [18,19]. One of the major challenges in addressing self-medication is the failure of pharmacies to adhere to their professional commitments. Their practices significantly contribute to the expansion of self-medication [20].

Self-medication has positive effects on individuals and healthcare systems when practiced correctly. However, its promotion needs a policy direction supported by scientific evidence specific to the local socio-economic and cultural contexts. Currently, systematic reviews and the recommendations or clinical practice guidelines (CPGs) provide the cornerstone of evidence-based medicine [21,22]. In Ethiopia, there are different systematic reviews considering self-medication practice. However, it is very difficult to provide the national self-medication status of the country from the general population so that this umbrella review will provide possibility to address a broad scope of issues related self-medication [22]. Therefore, this umbrella review is proposed to examine self-medication practice in Ethiopia to produce the best available evidence in the context of Ethiopia and help evidence-informed policymaking.

At present, based on our preliminary screening of the literature in PubMed and Google Scholar, and a search of registered systematic review databases such as PROSPERO, there are currently no umbrella reviews or published protocols examining the self-medication practices in Ethiopia.

### Study objectives

#### General objective.

The objective of this study is to identify the overall pooled estimate of self-medication practices and contributing factors in Ethiopia

#### Specific objectives.

To examine self-medication practices in Ethiopia
To identify factors associated with self-medication practices

### Review questions

The question of this review include:

What are the experiences/meaningfulness/appropriateness of self-medication practice in Ethiopia?
What is the magnitude of self-medication in Ethiopia?
What are the factors contributing to self-medication in Ethiopia?

### Inclusion criteria

#### Participants.

This umbrella review will include systematic reviews reporting self-medication practices on adult population ( ≥ 18 years old) in Ethiopia. Systematic reviews with self-medication practices of families with their children less than 18 years will be excluded.

#### Intervention(s).

This umbrella review will include all systematic reviews that will evaluate self-medication practice. Self-medication is defined as the use of any drug or medication by individuals without the prescription, order, or supervision of a healthcare professional.

### Outcomes

This review will consider studies that include the following outcomes: the primary outcome is pooled magnitude of self-medication which is calculated as proportion of self-medication to total study participants. The secondary outcome will be factors associated with self-medication which is calculated in the form of two-by-two tables and reported using Odds ratio.

### Phenomena of interest

This review will include systematic reviews examining self-medication practices among any population group age ≥  18 years in Ethiopia.

### Context

All systematic reviews with or without meta-analysis in Ethiopia will be included in the current umbrella review.

### Types of research syntheses

This umbrella review will consider systematic review of all observational study designs with or without meta-analysis. The goal of JBI Umbrella Reviews is to assemble data from various study syntheses [22–24]. The review will also include qualitative syntheses of systematic review

## Methods

The planned review of umbrella review will be carried out using the JBI Umbrella review methodology [22,23]. The title for this review was registered on PROSPERO and obtained registration ID CRD42023182552.

### Search strategy

Comprehensive search including both published and unpublished research syntheses as per JBI Methodological recommendation [25] will be sought after using the search strategies. In this review, a three-step search methodology will be used. First, an initial limited search of MEDLINE (PubMed) and CINAHL (EBSCO) will be undertaken to identify articles on the topic. The text words contained in the titles and abstracts of relevant articles, and the index terms used to describe the articles will be used to develop a full search strategy for reporting the name of the relevant databases/information sources (S1 File). The search strategy, including all identified keywords and index terms, will be adapted for each included database and/or information source. The reference list of all included sources of evidence will be screened for additional studies.

Studies published in English language will be considered. All systematic review on self-medication practice in Ethiopia irrespective of year of publication fulfilling inclusion criteria will be included.

### Information sources

The databases to be searched for this review include MEDLINE (PubMed), Embase.com, CINAHL (EBSCO), and Web of Science. To capture unpublished reviews, additional searches will be conducted using Google Scholar and institutional websites, including those of the Ministry of Health (MoH), the Ethiopian Food and Drug Authority (EFDA), and Addis Ababa University (AAU).

### Study selection

Following the search, all identified citations will be collated and uploaded into EndNote 20 (Clarivate Analytics, PA, USA) and duplicates removed. After a pilot test, titles and abstracts will be reviewed by two independent reviewers to determine if they meet the inclusion requirements. Potentially pertinent articles will be located in their entirety, and their citation information imported into the JBI System for the Unified Management, Assessment and Review of Information (JBI SUMARI; The JBI, Adelaide, Australia) [22,26,27]. Two impartial reviewers will thoroughly examine the complete text of the chosen citations in comparison to the inclusion criteria. The umbrella review will keep track of and report on the factors that led to full-text articles being excluded when they failed to meet the inclusion criteria. Any disagreements that arise between the reviewers at each stage of the selection process will be resolved through discussion, or with a third reviewer. The complete search results will be provided in the final report and in Preferred Reporting Items for Systematic Reviews and Meta-analyses (PRISMA) [28].

### Assessment of methodological quality

Selected syntheses will be critically appraised by two independent reviewers (GF and TT) for methodological quality in the review using the standardized critical appraisal instrument from JBI [22]. Authors of papers will be contacted to request missing or additional data for clarification, where required. Any disagreements that arise between the reviewers will be resolved through discussion, or with a third reviewer. The results of the critical appraisal will be reported in narrative form and in a table. All syntheses, regardless of the results of their methodological quality, will undergo data extraction and synthesis.

### Data collection

Data will be extracted from syntheses included in the review by two independent reviewers using the standardized JBI data extraction tool [22]. The data extracted will include specific details about the populations, review methods, interventions, and outcomes of significance to the review objective. Any disagreements that arose between the reviewers were resolved through discussion or with a third reviewer. Authors of papers were contacted to request missing or additional data, where required. Any papers with missed information after two contacts of authors of the included studies will be excluded if no response obtained at after the second contact.

### Data summary

The above data extracted from selected reviews will be tabulated and accompanied by narrative synthesis to address the review objective and specific question. For quantitative systematic reviews included in the umbrella review, the number of studies that inform the outcome, the number of participants (from included studies), and the heterogeneity of the results of included reviews will be reported. For qualitative systematic reviews/systematic included in the umbrella review, the final or overall synthesized findings from included reviews will be presented.

The results of the umbrella review will be provided in tabular form in a “Summary of Evidence” table that includes reason, common illness, population groups using self-medication and common side effects.

### Assessing certainty in the outcome

The Grading of Recommendations, Assessment, Development and Evaluation (GRADE) approach for grading the certainty of evidence will be followed and a Summary of Findings (SoF) will be created using GRADEpro GDT [29] (McMaster University, ON, Canada). We will use the GRADE approach to determine how confident we are in the evidence from the reviews we include. The GRADE approach evaluates four aspects: the quality and design of the original studies, how consistent their results are, and how relevant they are to our question. We will rate the evidence as high, moderate, low, or very low quality. We will also summarize the findings for the five outcomes that our review focuses on: hospitalization, institutionalization, death, patient satisfaction, and quality of life [29,30]. This will be undertaken by two independent reviewers (GF and TT) at the outcome level. Any disagreements that arise between the reviewers will be resolved through discussion or with a third reviewer (SA). Authors of papers were contacted to request missing or additional data for clarification, where required through two emails request.

The SoF will present the following information where appropriate: absolute risks for the treatment and control, estimates of relative risk, and a ranking of the quality of the evidence based on the risk of bias, directness, heterogeneity, precision and risk of publication bias of the review results. The outcomes reported in the SoF will be self-medication practices in Ethiopia and factors associated with it as primary and secondary outcomes respectively.

### Assessing confidence in the synthesized findings

The final synthesized findings will be graded according to the ConQual approach for establishing confidence in the output of qualitative research synthesis and presented in a Summary of Findings [31]. The Summary of Findings includes the major elements of the review and details how the ConQual score is developed. Included in the Summary of Findings will be the title, population, phenomena of interest, and context for the specific review. Each synthesized finding from the review will then be presented, along with the type of research informing it, the score for dependability and credibility, and the overall ConQual score.

## Discussion

Antimicrobial resistance (AMR) is a global health threat closely linked to the inappropriate use of medications, including self-medication, which can diminish the effectiveness of antibiotics. The misuse and overuse of antimicrobials, particularly in humans, are among the primary drivers of drug-resistant pathogens. In recent years, the number of facilities providing medications has significantly increased in low-income countries like Ethiopia. Drug vendors, pharmacies, and wholesalers are now widespread in towns and cities, despite occasional shortages of essential medicines. However, reports frequently highlight that these facilities often sell medications without requiring prescriptions from authorized healthcare professionals, treating them like ordinary consumer goods. This practice exacerbates the global threat of antimicrobial resistance, as self-medication often leads to poor adherence to prescribed treatment regimens and incomplete courses of antibiotics, further exacerbating the development and spread of resistant pathogens.

As far as researchers’ knowledge is concerned this umbrella review is of its first kind in Ethiopia on self-medication practice. The review will focus to identify the overall pooled estimate of self-medication practices and contributing factors in Ethiopia. The result will give rich information to the evidence-based decisions making on self-medication practices in Ethiopia, which might be base to design policies and strategies to fight against anti-microbial and irrational use of medications like self-medication of different population segments within the country.

Any systematic review and/or Meta-analysis in Ethiopian context found both in published and unpublished form will be considered for better estimating the pooled magnitude of self-medication practices and its associated factors in Ethiopia. Thus, this umbrella review is needed to negotiate the result from systematic review and meta-analysis of self-medications found in Ethiopia. It is also crucial opportunity with study to state the status of self-medication practice and associated factors within Ethiopia. The study will also significantly important to embrace the factors that contribute to self-medication practice within Ethiopia, so that targeted intervention will be designed for each factor.

The result from this umbrella review will attract the attentions of government and partners for intervention of self-medication practices which is serving as driving engine to fight against anti-microbial resistance. Furthermore, it serves for policy makers by providing evidence on self-medication practices to design context specific intervention to halt self-medication practices. Parallel to this, it will supplement evidence for government to develop guidelines, policies and proclamations for use of self-medication practices which will play pivotal role in preventing anti-microbial resistance. The result form this review will essential for appropriate implementation of drug distribution and sells across all drug distrusting store, venders or whole sellers in Ethiopia, so that regulation of medication/drugs to augment effort to fight against global issue of AMR. It can be taken as a benchmark for future researchers undertaking their interventional and other high standard studies to reduce self-medication practices and anti-microbial resistances.

All efforts will be made to disseminate the evidence to scientific community through different means like present the work on different workshops, conference and publication on internationally reputable scientific journal. However, the two might be limitation of the review like related with language, and limitation of study area. This can cause bias on the outcome. Therefore, the finding of this review should use with caution to the other areas than within Ethiopia.

## Conclusion

The finding will used as a basic information for understanding the burden of self-medication in low-income countries like Ethiopia. Furthermore, this review will aid for design prevention of self-medication practice in Ethiopia as well as other country as antimicrobial resistance is one of the global concerns currently. In addition, the result will explore importance of self-medication in prevention of Global concerns like anti-microbial resistance.

## Supporting information

S1 File Database search result. (DOCX)

S2 File JBI quality appraisal tool. (DOCX)

S3 File PRISMA checklist. (DOC)
