# Supplementary material for: Self-medication practices in Ethiopia: An umbrella review protocol
Source: PLoS One. 2025 Feb 27;20(2):e0300131. doi: 10.1371/journal.pone.0300131 (PMC11867381; doi:10.1371/journal.pone.0300131)
Supplement: S1 File — (DOCX) [file pone.0300131.s001.docx]

# Appendices: Appendix I: Search strategy in Pubmed

| 14 | ((((((((((((((((((((((((Self-medication) OR (Drug Utilization)) OR (Non-prescription Drugs)) OR (Prescription Drugs)) OR (self-prescription)) OR (non-prescription)) OR (OTC drugs)) OR (over-the-counter medication)) OR (dispensing practice)) OR (Self Medicat∗)) OR (self-administration)) OR (illicit use)) OR (without doctor’s prescription)) OR (Nonprescription Drugs[Text Word])) OR (Drug Misuse)) OR (inappropriate)) OR (drug hoarding)) OR (self-treatment)) OR (OTC medicine used)) OR (taking the drug without order) AND (meta-analysis[Filter] OR systematicreview[Filter])) AND ((((((((General Population) OR (Pregnant mothers)) OR (Childbearing mothers)) OR (Child breaing womens)) OR (Health care providers)) OR (Health Professionals)) OR (University students)) OR (Adults age groups) AND (meta-analysis[Filter] OR systematicreview[Filter])) AND (meta-analysis[Filter] OR systematicreview[Filter])) AND (((Prevalence) OR (Epidemiology)) OR (Magnitude) AND (meta-analysis[Filter] OR systematicreview[Filter])) AND (meta-analysis[Filter] OR systematicreview[Filter])) OR ((((Associated factor) OR (Determinants)) OR (Predictors)) OR (risk factors) AND (meta-analysis[Filter] OR systematicreview[Filter])) AND (meta-analysis[Filter] OR systematicreview[Filter])) AND (((Systematic review) OR (Review)) OR (Meta-analysis) AND (meta-analysis[Filter] OR systematicreview[Filter])) AND (meta-analysis[Filter] OR systematicreview[Filter])) AND (Ethiopia) AND (meta-analysis[Filter] OR systematicreview[Filter]) | 1,535 |
| --- | --- | --- |
| 13 | ((((((((((((((((((((((((Self-medication) OR (Drug Utilization)) OR (Non-prescription Drugs)) OR (Prescription Drugs)) OR (self-prescription)) OR (non-prescription)) OR (OTC drugs)) OR (over-the-counter medication)) OR (dispensing practice)) OR (Self Medicat∗)) OR (self-administration)) OR (illicit use)) OR (without doctor’s prescription)) OR (Nonprescription Drugs[Text Word])) OR (Drug Misuse)) OR (inappropriate)) OR (drug hoarding)) OR (self-treatment)) OR (OTC medicine used)) OR (taking the drug without order) AND (meta-analysis[Filter] OR systematicreview[Filter])) AND ((((((((General Population) OR (Pregnant mothers)) OR (Childbearing mothers)) OR (Child breaing womens)) OR (Health care providers)) OR (Health Professionals)) OR (University students)) OR (Adults age groups) AND (meta-analysis[Filter] OR systematicreview[Filter])) AND (meta-analysis[Filter] OR systematicreview[Filter])) AND (((Prevalence) OR (Epidemiology)) OR (Magnitude) AND (meta-analysis[Filter] OR systematicreview[Filter])) AND (meta-analysis[Filter] OR systematicreview[Filter])) OR ((((Associated factor) OR (Determinants)) OR (Predictors)) OR (risk factors) AND (meta-analysis[Filter] OR systematicreview[Filter])) AND (meta-analysis[Filter] OR systematicreview[Filter])) AND (((Systematic review) OR (Review)) OR (Meta-analysis) AND (meta-analysis[Filter] OR systematicreview[Filter])) AND (meta-analysis[Filter] OR systematicreview[Filter])) AND (Ethiopia) AND (meta-analysis[Filter] OR systematicreview[Filter]) | 1,535 |
| 12 | ((((((((((((((((((((((((Self-medication) OR (Drug Utilization)) OR (Non-prescription Drugs)) OR (Prescription Drugs)) OR (self-prescription)) OR (non-prescription)) OR (OTC drugs)) OR (over-the-counter medication)) OR (dispensing practice)) OR (Self Medicat∗)) OR (self-administration)) OR (illicit use)) OR (without doctor’s prescription)) OR (Nonprescription Drugs[Text Word])) OR (Drug Misuse)) OR (inappropriate)) OR (drug hoarding)) OR (self-treatment)) OR (OTC medicine used)) OR (taking the drug without order) AND (meta-analysis[Filter] OR systematicreview[Filter])) AND ((((((((General Population) OR (Pregnant mothers)) OR (Childbearing mothers)) OR (Child breaing womens)) OR (Health care providers)) OR (Health Professionals)) OR (University students)) OR (Adults age groups) AND (meta-analysis[Filter] OR systematicreview[Filter])) AND (meta-analysis[Filter] OR systematicreview[Filter])) AND (((Prevalence) OR (Epidemiology)) OR (Magnitude) AND (meta-analysis[Filter] OR systematicreview[Filter])) AND (meta-analysis[Filter] OR systematicreview[Filter])) OR ((((Associated factor) OR (Determinants)) OR (Predictors)) OR (risk factors) AND (meta-analysis[Filter] OR systematicreview[Filter])) AND (meta-analysis[Filter] OR systematicreview[Filter])) AND (((Systematic review) OR (Review)) OR (Meta-analysis) AND (meta-analysis[Filter] OR systematicreview[Filter])) AND (meta-analysis[Filter] OR systematicreview[Filter])) AND (Ethiopia) AND (meta-analysis[Filter] OR systematicreview[Filter]) | 1,535 |
| 11 | ((((((((((((((((((((((((Self-medication) OR (Drug Utilization)) OR (Non-prescription Drugs)) OR (Prescription Drugs)) OR (self-prescription)) OR (non-prescription)) OR (OTC drugs)) OR (over-the-counter medication)) OR (dispensing practice)) OR (Self Medicat∗)) OR (self-administration)) OR (illicit use)) OR (without doctor’s prescription)) OR (Nonprescription Drugs[Text Word])) OR (Drug Misuse)) OR (inappropriate)) OR (drug hoarding)) OR (self-treatment)) OR (OTC medicine used)) OR (taking the drug without order) AND (meta-analysis[Filter] OR systematicreview[Filter])) AND ((((((((General Population) OR (Pregnant mothers)) OR (Childbearing mothers)) OR (Child breaing womens)) OR (Health care providers)) OR (Health Professionals)) OR (University students)) OR (Adults age groups) AND (meta-analysis[Filter] OR systematicreview[Filter])) AND (meta-analysis[Filter] OR systematicreview[Filter])) AND (((Prevalence) OR (Epidemiology)) OR (Magnitude) AND (meta-analysis[Filter] OR systematicreview[Filter])) AND (meta-analysis[Filter] OR systematicreview[Filter])) OR ((((Associated factor) OR (Determinants)) OR (Predictors)) OR (risk factors) AND (meta-analysis[Filter] OR systematicreview[Filter])) AND (meta-analysis[Filter] OR systematicreview[Filter])) AND (((Systematic review) OR (Review)) OR (Meta-analysis) AND (meta-analysis[Filter] OR systematicreview[Filter])) AND (meta-analysis[Filter] OR systematicreview[Filter])) AND (Ethiopia) AND (meta-analysis[Filter] OR systematicreview[Filter]) | 1,535 |
| 10 | ((((((((((((((((((((((((Self-medication) OR (Drug Utilization)) OR (Non-prescription Drugs)) OR (Prescription Drugs)) OR (self-prescription)) OR (non-prescription)) OR (OTC drugs)) OR (over-the-counter medication)) OR (dispensing practice)) OR (Self Medicat∗)) OR (self-administration)) OR (illicit use)) OR (without doctor’s prescription)) OR (Nonprescription Drugs[Text Word])) OR (Drug Misuse)) OR (inappropriate)) OR (drug hoarding)) OR (self-treatment)) OR (OTC medicine used)) OR (taking the drug without order) AND (meta-analysis[Filter] OR systematicreview[Filter])) AND ((((((((General Population) OR (Pregnant mothers)) OR (Childbearing mothers)) OR (Child breaing womens)) OR (Health care providers)) OR (Health Professionals)) OR (University students)) OR (Adults age groups) AND (meta-analysis[Filter] OR systematicreview[Filter])) AND (meta-analysis[Filter] OR systematicreview[Filter])) AND (((Prevalence) OR (Epidemiology)) OR (Magnitude) AND (meta-analysis[Filter] OR systematicreview[Filter])) AND (meta-analysis[Filter] OR systematicreview[Filter])) OR ((((Associated factor) OR (Determinants)) OR (Predictors)) OR (risk factors) AND (meta-analysis[Filter] OR systematicreview[Filter])) AND (meta-analysis[Filter] OR systematicreview[Filter])) AND (((Systematic review) OR (Review)) OR (Meta-analysis) AND (meta-analysis[Filter] OR systematicreview[Filter])) AND (meta-analysis[Filter] OR systematicreview[Filter])) AND (Ethiopia) | 1,535 |
| 9 | (((((((((((((((((((((((Self-medication) OR (Drug Utilization)) OR (Non-prescription Drugs)) OR (Prescription Drugs)) OR (self-prescription)) OR (non-prescription)) OR (OTC drugs)) OR (over-the-counter medication)) OR (dispensing practice)) OR (Self Medicat∗)) OR (self-administration)) OR (illicit use)) OR (without doctor’s prescription)) OR (Nonprescription Drugs[Text Word])) OR (Drug Misuse)) OR (inappropriate)) OR (drug hoarding)) OR (self-treatment)) OR (OTC medicine used)) OR (taking the drug without order) AND (meta-analysis[Filter] OR systematicreview[Filter])) AND ((((((((General Population) OR (Pregnant mothers)) OR (Childbearing mothers)) OR (Child breaing womens)) OR (Health care providers)) OR (Health Professionals)) OR (University students)) OR (Adults age groups) AND (meta-analysis[Filter] OR systematicreview[Filter])) AND (meta-analysis[Filter] OR systematicreview[Filter])) AND (((Prevalence) OR (Epidemiology)) OR (Magnitude) AND (meta-analysis[Filter] OR systematicreview[Filter])) AND (meta-analysis[Filter] OR systematicreview[Filter])) OR ((((Associated factor) OR (Determinants)) OR (Predictors)) OR (risk factors) AND (meta-analysis[Filter] OR systematicreview[Filter])) AND (meta-analysis[Filter] OR systematicreview[Filter])) AND (((Systematic review) OR (Review)) OR (Meta-analysis) AND (meta-analysis[Filter] OR systematicreview[Filter])) | 2,74,583 |
| 8 | ((((((((((((((((((((((Self-medication) OR (Drug Utilization)) OR (Non-prescription Drugs)) OR (Prescription Drugs)) OR (self-prescription)) OR (non-prescription)) OR (OTC drugs)) OR (over-the-counter medication)) OR (dispensing practice)) OR (Self Medicat∗)) OR (self-administration)) OR (illicit use)) OR (without doctor’s prescription)) OR (Nonprescription Drugs[Text Word])) OR (Drug Misuse)) OR (inappropriate)) OR (drug hoarding)) OR (self-treatment)) OR (OTC medicine used)) OR (taking the drug without order) AND (meta-analysis[Filter] OR systematicreview[Filter])) AND ((((((((General Population) OR (Pregnant mothers)) OR (Childbearing mothers)) OR (Child breaing womens)) OR (Health care providers)) OR (Health Professionals)) OR (University students)) OR (Adults age groups) AND (meta-analysis[Filter] OR systematicreview[Filter])) AND (meta-analysis[Filter] OR systematicreview[Filter])) AND (((Prevalence) OR (Epidemiology)) OR (Magnitude) AND (meta-analysis[Filter] OR systematicreview[Filter])) AND (meta-analysis[Filter] OR systematicreview[Filter])) OR ((((Associated factor) OR (Determinants)) OR (Predictors)) OR (risk factors) AND (meta-analysis[Filter] OR systematicreview[Filter])) | 2,74,583 |
| 7 | (((((((((((((((((((((Self-medication) OR (Drug Utilization)) OR (Non-prescription Drugs)) OR (Prescription Drugs)) OR (self-prescription)) OR (non-prescription)) OR (OTC drugs)) OR (over-the-counter medication)) OR (dispensing practice)) OR (Self Medicat∗)) OR (self-administration)) OR (illicit use)) OR (without doctor’s prescription)) OR (Nonprescription Drugs[Text Word])) OR (Drug Misuse)) OR (inappropriate)) OR (drug hoarding)) OR (self-treatment)) OR (OTC medicine used)) OR (taking the drug without order) AND (meta-analysis[Filter] OR systematicreview[Filter])) AND ((((((((General Population) OR (Pregnant mothers)) OR (Childbearing mothers)) OR (Child breaing womens)) OR (Health care providers)) OR (Health Professionals)) OR (University students)) OR (Adults age groups) AND (meta-analysis[Filter] OR systematicreview[Filter])) AND (meta-analysis[Filter] OR systematicreview[Filter])) AND (((Prevalence) OR (Epidemiology)) OR (Magnitude) AND (meta-analysis[Filter] OR systematicreview[Filter])) | 978 |
| 6 | ((((((((((((((((((((Self-medication) OR (Drug Utilization)) OR (Non-prescription Drugs)) OR (Prescription Drugs)) OR (self-prescription)) OR (non-prescription)) OR (OTC drugs)) OR (over-the-counter medication)) OR (dispensing practice)) OR (Self Medicat∗)) OR (self-administration)) OR (illicit use)) OR (without doctor’s prescription)) OR (Nonprescription Drugs[Text Word])) OR (Drug Misuse)) OR (inappropriate)) OR (drug hoarding)) OR (self-treatment)) OR (OTC medicine used)) OR (taking the drug without order) AND (meta-analysis[Filter] OR systematicreview[Filter])) AND ((((((((General Population) OR (Pregnant mothers)) OR (Childbearing mothers)) OR (Child breaing womens)) OR (Health care providers)) OR (Health Professionals)) OR (University students)) OR (Adults age groups) AND (meta-analysis[Filter] OR systematicreview[Filter])) | 2,803 |
| 5 | ((Systematic review) OR (Review)) OR (Meta-analysis) | 3,54,949 |
| 4 | (((((((General Population) OR (Pregnant mothers)) OR (Childbearing mothers)) OR (Child breaing womens)) OR (Health care providers)) OR (Health Professionals)) OR (University students)) OR (Adults age groups) | 1,00,215 |
| 3 | (((Associated factor) OR (Determinants)) OR (Predictors)) OR (risk factors) | 2,74,359 |
| 2 | ((Prevalence) OR (Epidemiology)) OR (Magnitude) | 1,08,649 |
| 1 | (((((((((((((((((((Self-medication) OR (Drug Utilization)) OR (Non-prescription Drugs)) OR (Prescription Drugs)) OR (self-prescription)) OR (non-prescription)) OR (OTC drugs)) OR (over-the-counter medication)) OR (dispensing practice)) OR (Self Medicat∗)) OR (self-administration)) OR (illicit use)) OR (without doctor’s prescription)) OR (Nonprescription Drugs[Text Word])) OR (Drug Misuse)) OR (inappropriate)) OR (drug hoarding)) OR (self-treatment)) OR (OTC medicine used)) OR (taking the drug without order) | 7,100 |
