# Supplementary material for: Self-medication practices in Ethiopia: An umbrella review protocol
Source: PLoS One. 2025 Feb 27;20(2):e0300131. doi: 10.1371/journal.pone.0300131 (PMC11867381; doi:10.1371/journal.pone.0300131)
Supplement: S2 File — (DOCX) [file pone.0300131.s002.docx]

**Appendix II: The JBI data extraction instrument**

| **Study Details** |  |
| --- | --- |
| Author/year |  |
| objectives |  |
| Participants (characteristics/total  number) |  |
| Setting/context |  |
| Description of Interventions/  phenomena of interest |  |
| **Search Details** |  |
| Sources searched |  |
| Range (years) of included studies |  |
| Number of studies included / Types of studies included |  |
| Country of origin of incl. studies |  |
| **Appraisal** |  |
| Appraisal instruments used |  |
| Appraisal rating |  |
| **Analysis** |  |
| Method of analysis |  |
| Outcome assessed |  |
| Results/Findings |  |
| Significance/direction |  |
| Heterogeneity |  |
| Comments |  |
